# Fused in Liposarcoma Protein, a New Player in the Regulation of HIV-1 Transcription, Binds to Known and Newly Identified LTR G-Quadruplexes

**DOI:** 10.1021/acsinfecdis.1c00508

**Published:** 2022-05-03

**Authors:** Emanuela Ruggiero, Ilaria Frasson, Elena Tosoni, Matteo Scalabrin, Rosalba Perrone, Maja Marušič, Janez Plavec, Sara N. Richter

**Affiliations:** † Department of Molecular Medicine, University of Padua, via Aristide Gabelli 63, Padua 35121, Italy; ‡ Buck Institute for Research on Aging, 8001 Redwood Boulevard, Novato, California 94945, United States; § Slovenian NMR Center, National Institute of Chemistry, Hajdrihova, 19, Ljubljana SI-1000, Slovenia

**Keywords:** HIV-1, G-quadruplex, FUS, LTR promoter, viral transcription

## Abstract

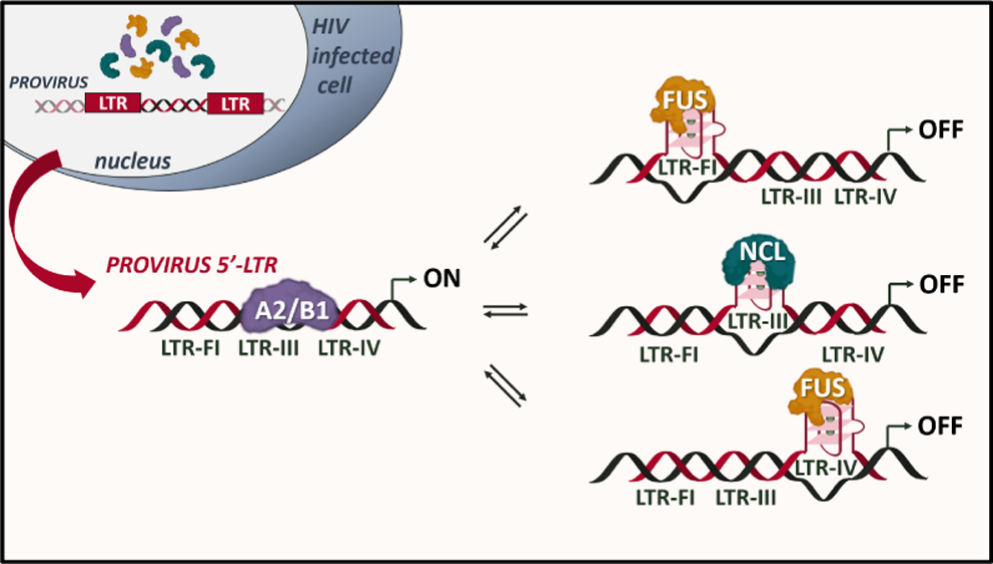

HIV-1 integrated long terminal repeat (LTR) promoter activity is modulated by folding of its G-rich region into non-canonical nucleic acids structures, such as G-quadruplexes (G4s), and their interaction with cellular proteins. Here, by a combined pull-down/mass spectrometry/Western-blot approach, we identified the fused in liposarcoma (FUS) protein and found it to preferentially bind and stabilize the least stable and bulged LTR G4, especially in the cell environment. The outcome of this interaction is the down-regulation of viral transcription, as assessed in a reporter assay with LTR G4 mutants in FUS-silencing conditions. These data indicate that the complexity and dynamics of HIV-1 LTR G4s are much greater than previously envisaged. The G-rich LTR region, with its diverse G4 landscape and multiple cell protein interactions, stands out as prime sensing center for the fine regulation of viral transcription. This region thus represents a rational antiviral target for inhibiting both the actively transcribing and latent viruses.

The human immunodeficiency virus type 1 (HIV-1) is the retrovirus responsible for the acquired immunodeficiency syndrome, today affecting more than 38 million people worldwide.^1^ HIV-1 establishes a life-long latent infection by integrating a double-stranded (ds)-DNA copy of its diploid RNA viral genome into the host cell’s DNA. The currently available antiretroviral therapy, which consists of a combination of three or more drugs with different mechanisms of action, is highly efficient in keeping disease progression under control,^2^ but it does not target the integrated genome (provirus); hence, to date there are no feasible strategies to eradicate the virus from its host. Consequently, the fight against HIV-1 is still a major challenge for the scientific community that strives to disclose novel pathogenic mechanisms to identify new possible antiviral targets.

G-quadruplexes (G4s) are non-canonical nucleic acid secondary structures that may form in guanine (G)-rich strands when two or more G-tetrads stack on top of each other coordinated by monovalent cations (Figure 1A).^3^ G4s are mainly located in genomic regulatory regions, like telomeres, oncogene promoters, and recombination sites, where they have been widely reported to regulate pivotal cellular processes, such as genome replication, transcription, translation, DNA damage response, and many others.^4^ G4 biological roles have been assessed not only in humans but also in other organisms like bacteria,^5^ yeasts,^6^ and viruses.^7^ Putative G4-forming sequences (PQSs), characterized by high conservation rates among strains and statistically significant distribution within the virus genome, have been reported in almost all human viruses.^8,9^ Considering the typically high virus genome variability, the observed PQS conservation strongly supports a crucial role of G4s in viruses. Functional studies proved G4 formation at distinctive genomic regions in both DNA and RNA viruses and their influence at different steps of the viral life cycle. For example, in the Herpes simplex virus type-1 (HSV-1), a DNA virus, G4s are located in repeated regions and in immediate early gene promoters:^10−13^ G4s fold extensively in the nucleus during viral replication and later migrate toward the membrane, to be finally found in newly released virions.^14,15^ Treatment with G4 ligands reduced viral replication, impairing viral DNA synthesis.^10,15,16^ Among RNA viruses, G4s in hepatitis C virus (HCV), which has a (+)ss-RNA genome, have been reported by several groups. HCV genome replication was shown to be regulated by highly conserved G4s located in the core gene, through interaction with cellular proteins. G4 ligands hampered genomic RNA synthesis and disrupted G4/protein interaction, affecting the cellular antiviral immune response.^17,18^ Similar investigations have been conducted in different viruses, such as the human papillomavirus (HPV), most *Herpesviridae* family members, the hepatitis B virus (HBV), and flavi-, filo-, and retroviruses, and research keeps blooming in the field, bolstering G4’s crucial role in viruses and G4 targeting for antiviral therapy.^7,19^

**Figure 1:**
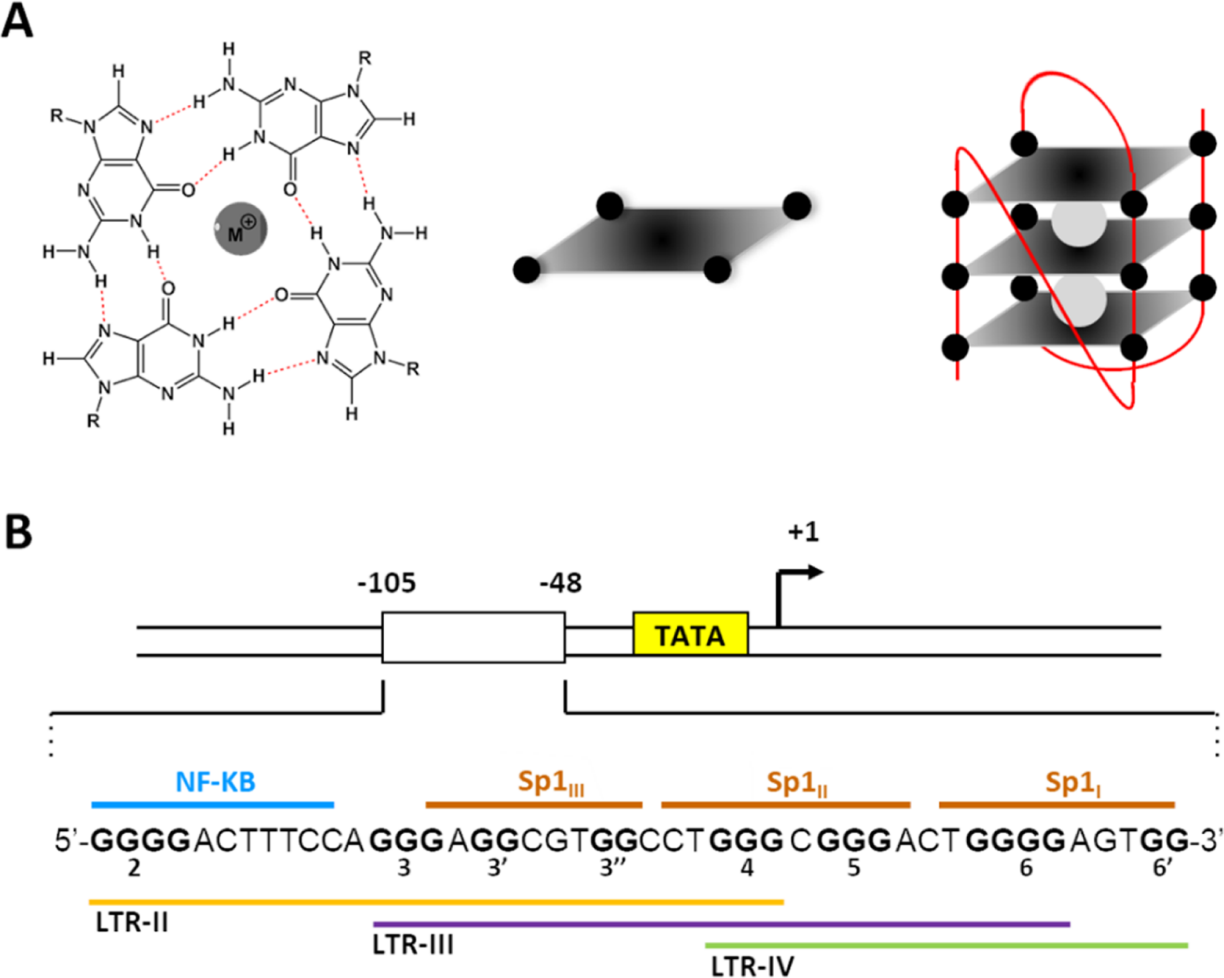
G4 landscape in the HIV-1 LTR promoter. (A) Four Gs linked together through Hoogsteen-type H-bonds are coordinated by monovalent cations to form a G-tetrad; multiple tetrads self-stack to give the quadruplex structure. (B) The HIV-1 LTR includes three overlapping G4-forming sequences, encompassing one NF-κB and three SP1 TF binding sites.

In the cellular context, G4s are regulated by the interaction with proteins that either stabilize or unfold them. In the past few years, research in this field has led to the identification of a diverse set of proteins able to interact with G4s at both the DNA and RNA levels.^20^ Some proteins, such as nucleolin (NCL), have been reported to stabilize G4s in cells^21^ and virus^18,22^ promoters and impair transcription; others, like several helicases and human ribonucleoproteins (hnRNPs), in contrast, have been shown to hamper G4 folding and promote polymerase progression.^23,24^

In HIV-1, we had previously shown that the virus long terminal promoter region (LTR) is regulated by three dynamic and mutually exclusive G4s, namely, LTR-II, LTR-III, and LTR-IV (Figure 1B).^25^ LTR-III is the major G4 that folds in the G-rich LTR promoter sequence: it is formed by a three-layered hybrid conformation G4 and a stem loop with Watson and Crick G/C base pairing in the long loop.^26^ LTR-IV folds into a parallel-stranded G4 with a bulged thymine (T) residue that is involved in a conserved stacking interaction with the adenine (A) nucleotide located in the loop.^27^ LTR-IV G4 acts as a negative regulator of LTR-III G4: its folding is weaker than that of LTR-III but inducible upon binding by other agents, such as ligands or proteins.^22,28^ HIV-1 LTR G4-forming sequences encompass NF-κB and SP1 transcription factor (TF) binding sites: this feature is common to all primate lentiviruses, the genus that HIV-1 belongs to, making G4s essential and conserved-throughout-evolution virus regulatory elements.^9^ We proved that the LTR G/C-rich region is processed by several cellular proteins: NCL, which mainly stabilizes LTR-III G4;^22^ hnRNP A2/B1, which hinders LTR G4 formation;^29^ and hnRNP K, which induces i-motif folding in the LTR C-rich reverse strand.^30^ Together, these cellular proteins modulate viral transcription, through the fine tuning of non-canonical DNA structures at the provirus promoter.

To further characterize HIV-1 at the G4 level, here, we performed a combined pull-down/mass spectrometry (MS) analysis that led to the identification of the translocated/fused in liposarcoma (FUS) cellular protein as an additional HIV-1 LTR G4 binding protein. We showed that FUS binds to and stabilizes LTR G4s, with specific induction of a newly identified LTR G4 structure. The outcome of this interaction was negative modulation of viral transcription.

## Results

### Cellular Protein FUS Binds to HIV-1 LTR-IV G4

To extend our knowledge on HIV-1 LTR G4-mediated transcription regulation, here we sought to identify cellular proteins able to interact with and possibly promote the folding of LTR-IV G4. To this end, we adopted a combined pull-down/MS strategy: the biotinylated LTR-IV oligonucleotide and two negative control oligonucleotides unable to fold into G4 (LTR-IV random and LTR-IV MUT, Table 1) were incubated with nuclear extracts from HEK 293T cells, which are susceptible and permissive to HIV-1 infection.^31^ The proteins bound to the baits were subjected to SDS-PAGE/MS: data were analyzed by Mascot software, which assigns a score based on the number of fragments that match the recognized protein and the probability that the observed match is not a random event. The experiment was performed in 4 biological replicates: in each instance, only one protein, FUS, reported a score of >100 and displayed a high selectivity toward the G4 structure (i.e., score on the G4 > 4 times higher than the score on the negative control sequences). The complete list of proteins bound to the studied G4 sequence and with negligible affinity for the negative control sequence is reported in Table 1.

**Table 1:** Proteins Recovered in the Pull-Down/MS Analysis with the LTR-VI G4 Bait^a^

G4 bait	protein acronym	protein name (UniProt)	score	cellular localization
LTR-IV	FUS_HUMAN	RNA-binding protein FUS	419	nucleus
	PSPC1_HUMAN	paraspeckle component 1	85	nucleus
				nucleus matrix
				nuclear speckle
	NONO_HUMAN	non-POU domain-containing octamer-binding protein	65	nucleus
				nucleolus
				nuclear speckle
	XRCC6_HUMAN	X-ray repair cross-complementing protein 6	63	nucleus
	RBM39_HUMAN	RNA-binding protein 39	61	nuclear speckles
				core spliceosomal snRNP proteins

a Protein matches were obtained in two independent experiments. LTR-IV was used as the G4 bait, while a randomly composed oligonucleotide unable to fold into G4 (LTR-IV random in Table 2) was used as the control. Protein hits with scores lower than 30 were not retained nor those displaying scores higher than 30 in the interaction with the magnetic streptavidin-coated matrix. The score values were assigned by Mascot software and indicate the probability that the observed protein match is not a random event, and they are strictly related to the number of fragments that match the indicated protein.

### FUS Binds to Viral HIV-1 LTR G4s

**Figure 2:**
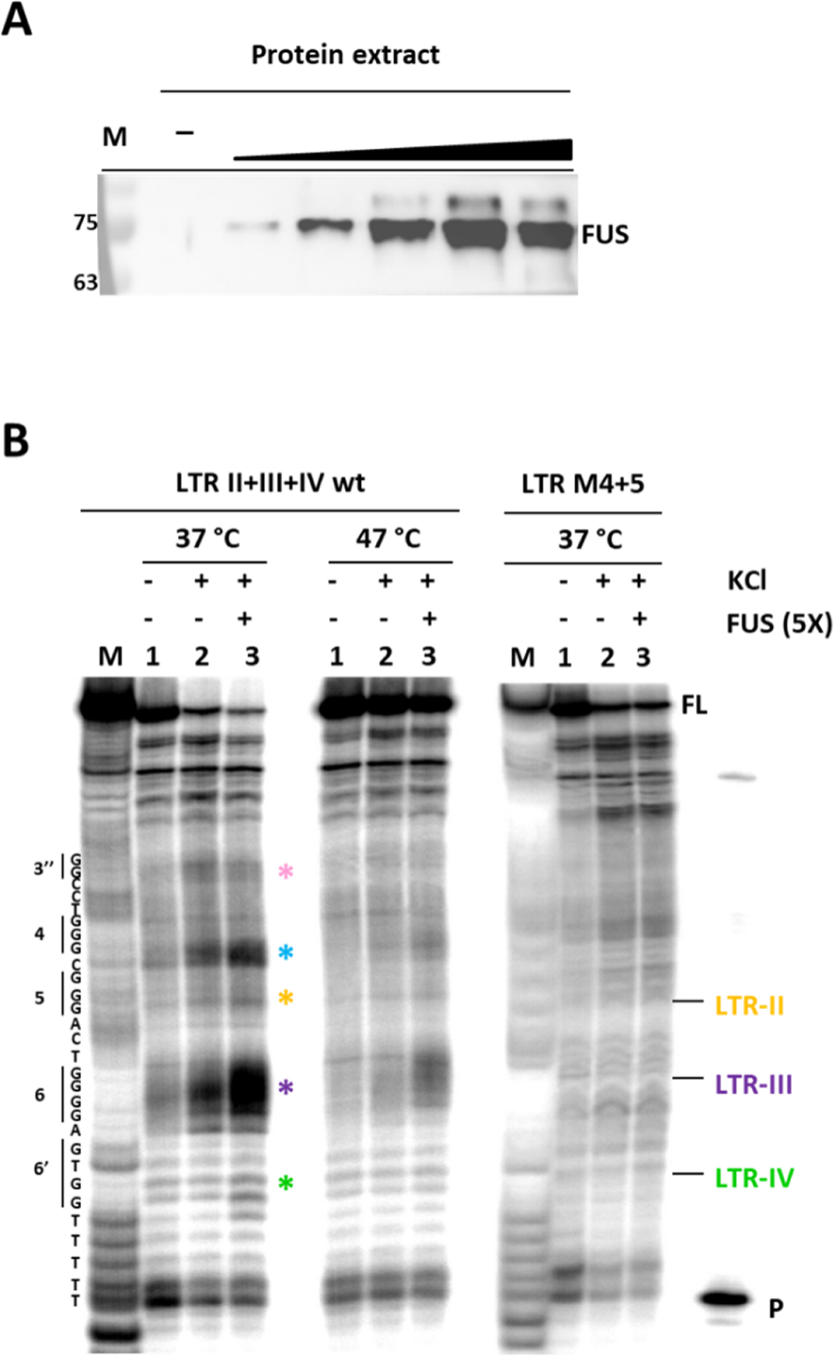
In vitro characterization of FUS binding to HIV-1 LTR G4s. (A) Pull-down/WB analysis of LTR-IV with the native FUS protein from cell extracts at increasing concentrations. (B) *Taq* polymerase stop assay was performed in the absence and presence of KCl 100 mM and FUS on the wt and mutant LTR-II + III + IV sequences. Amplification of the wt template was performed at 37 and 47 °C, as indicated; analysis of the mutant template was conducted at 37 °C. M indicates a marker lane obtained through the Maxam and Gilbert protocol; P indicates the primer; asterisks indicate K^+^- and/or FUS-induced stop bands.

FUS is a multifunctional RNP, involved in the regulation of key cellular processes like transcription, mRNA splicing, and transportation into the cytoplasm.^23,24^ To confirm LTR-IV/FUS binding, we performed the pull-down/Western-blot (WB) analysis in the presence of increasing concentrations of cell nuclear extracts containing the native protein (Figure 2A). Binding was concentration-dependent until saturation (Figure S1C). The amount of FUS protein in cell extracts was calculated comparing WB bands with those of a purified recombinant protein. Apparent *K*_D_ for LTR-IV/FUS interaction was 1.74 μM (Figure S1). Folding of the biotinylated LTR-IV G4 was confirmed by the circular dichroism (CD) analysis (Figure S2A).

We next extended the FUS binding analysis to the complete LTR G-rich sequence, that is, LTR-II + III + IV, by performing the *Taq* polymerase stop assay. G4 folding and FUS binding would hinder enzyme progression during elongation, with the consequent formation of truncated products that can be visualized in denaturing PAGE.^25^ After annealing to the G4 template, primer elongation was conducted at 37 °C (physiological temperature) and 47 °C (G4-destabilizing temperature), in the presence of KCl 100 mM, with and without FUS (5-molar excess) (Figure 2B). A control in the absence of K^+^ was used to obtain the band background produced by random pausing of the enzyme and possible template degradation. A mutated sequence (LTR-M4 + 5, Table 2) unable to fold into G4 was used alongside as the negative control (Figure S2B).^22,29^ In G4-inducing conditions (lanes 2) at 37 °C, increased stop bands corresponding to G-tracts 6, 5, 4, and 3″, which are involved in LTR-III and LTR-II G4s, were visible. As expected, bands corresponding to G-tract 6′, which is diagnostic for LTR-IV G4 formation, were not increased with respect to control lane 1.^25^ The observed truncated products, which were detected only when elongation was performed at 37 °C, confirmed the dynamic behavior of LTR G4s. Upon the addition of recombinant FUS (lanes 3) at 37 °C, all stop band signals intensified, including those at G-tract 6′, corresponding to LTR-IV; at 47 °C, only stop bands at G-tracts 6 and 4 remained visible, indicating that these Gs are involved in the tighter binding with the protein. No pausing was observed in any conditions on the negative control sequence LTR-M4 + 5. Because the most intense stop bands are generally observed at the G-tract at the 3′-end of the sequence involved in G4 folding, these data show that FUS at physiological temperatures binds to and stabilizes LTR-IV, LTR-III, LTR-II, and also an additional G4 not previously reported (G-tract 4, blue asterisk in Figure 2B); LTR-III and the new G4 are the most tightly bound because they are maintained at 47 °C. Note that in previous experiments conducted on the same template in the presence of NCL and hnRNPA2/B1 proteins, stop bands corresponding to LTR FUS-induced (FI) were never observed,^22,29^ corroborating FUS selectivity for the newly identified G4.

**Table 2:** List of All Oligonucleotides Used in This Study

assay	name	sequence (5′-3′)
pull-down	LTR-IV	TTTTTGGGCGGGACTGGGGAGTGGTTTTT[BtnTg]
	LTR-IV random	AAAACAGACAGCTCACTGCGCTCACAAAA[BtnTg]
	LTR-IV MUT	TTTTTGGGCGGGACTGGGTAGTGGTTTTT[BtnTg]
	LTR-FI 5T	TTTTTGGGGACTTTCCAGGGAGGCGTGGCCTGGGTTTTT[BtnTg]
	LTR-FI no T	GGGGACTTTCCAGGGAGGCGTGGCCTGGG[BtnTg]
	LTR-III	TTTTTGGGAGGCGTGGCCTGGGCGGGACTGGGGTTTTT[BtnTg]
	LTR-II + III + IV 5T	TTTTTGGGGACTTTCCAGGGAGGCGTGGCCTGGGCGGGACTGGGGAGTGGTTTTT[BtnTg]
	LTR-II + III + IV no T	GGGGACTTTCCAGGGAGGCGTGGCCTGGGCGGGACTGGGGAGTGG [BtnTg]
	LTR-II + III + IV random	AAAAACTACTGCACGCTCGCTACGACGACACTGTCGCGATACAAGCTGCAAAAA[BtnTg]
	G-rich	AGAGTGAGAGTGAGAGTGAGAGTG[BtnTg]
*Taq* pol stop	LTR-II + III + IV Taq	TTTTTGGGGACTTTCCAGGGAGGCGTGGCCTGGGCGGGACTGGGGAGTGGTTTTTCTGCATATAAGCAGCTGCTTTTTGCC
	LTR M4 + 5 Taq	TTTTTGGGGACTTTCCAGGGAGGCGTGGCCTGTGCGTGACTGGGGAGTGGTTTTTCTGCATATAAGCAGCTGCTTTTTGCC
	Taq primer	GGCAAAAAGCAGCTGCTTATATGCAG
CD 1H NMR	LTR-FI	GGGGACTTTCCAGGGAGGCGTGGCCTGGG
	LTR-II + III + IV	TTTTTGGGGACTTTCCAGGGAGGCGTGGCCTGGGCGGGACTGGGGAGTGGTTTTT
	LTR M4 + 5	TTTTTGGGGACTTTCCAGGGAGGCGTGGCCTGTGCGTGACTGGGGAGTGGTTTTT

**Figure 3:**
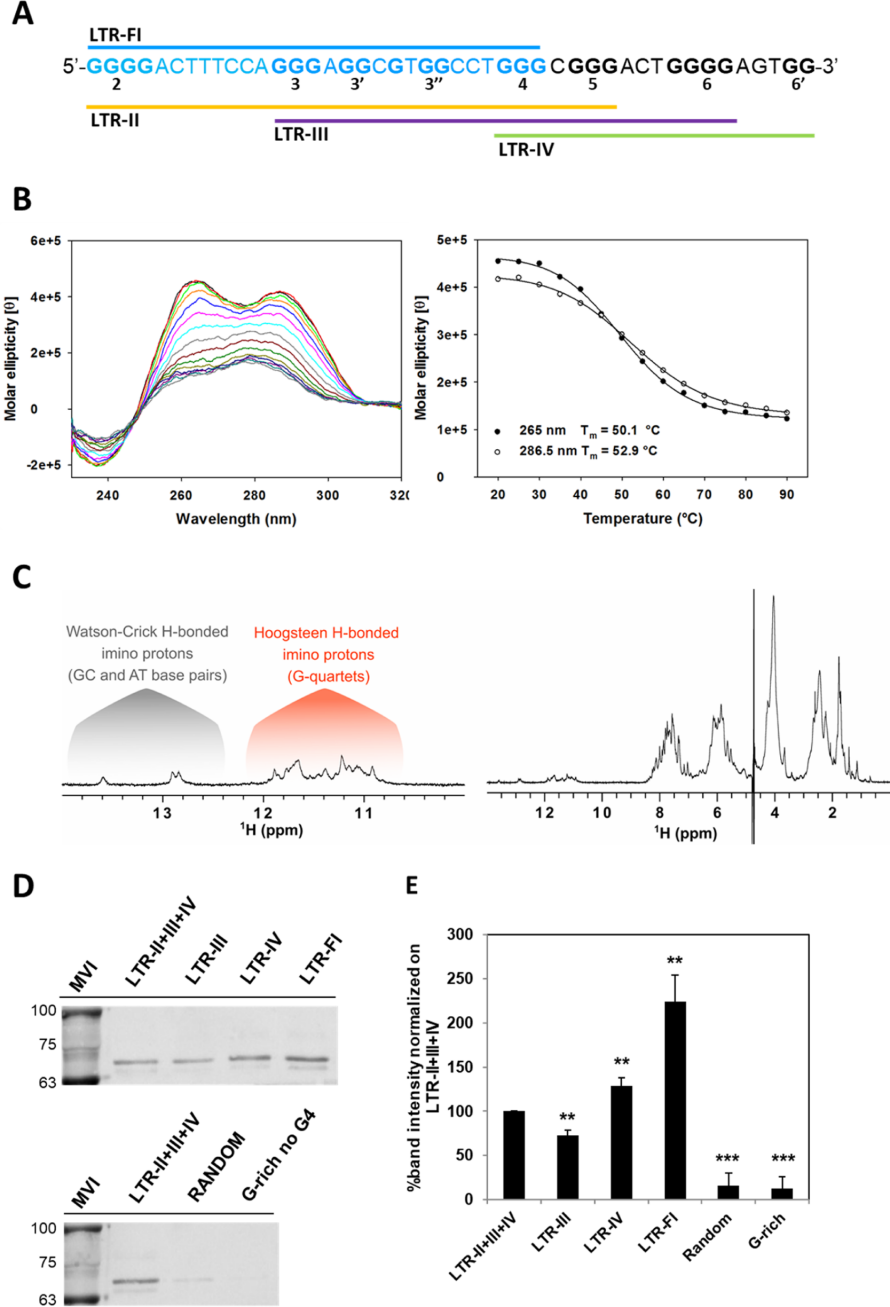
FUS binding to HIV-1 LTR-FI G4. (A) The newly identified HIV-1 LTR-FI G4 sequence is shown in blue within the full-length G-rich LTR region. (B) Representative CD spectrum of LTR-FI G4 (left panel) and relative melting profile measured at the two wavelengths corresponding to positive CD spectrum peaks (right panel). [θ] = deg·cm^2^/dmol. (C) 1D ^1^H NMR spectrum of LTR-FI (right panel) and expanded imino region (left panel). The spectrum was recorded at 25 °C, in 20 mM phosphate buffer pH 7.4, 80 mM KCl, and 1 mM oligonucleotide concentration. (D) WB analysis with the anti-FUS antibody, following pull-down and cross-linking. Bound fractions are reported for LTR G4s (upper panel) and negative control sequences (lower panel). MVI is the molecular weight ladder lane. (E) WB band quantification reported as a percentage relative to LTR-II + III + IV G4. **P < 0.05 ***P < 0.01.

We hypothesized LTR-FI G4 to be formed by three Gs from each of the G-tracts 2, 3, and 4 and by three additional Gs from the sequence comprised between G-tracts 3′ and 3″ (Figure 3A). CD analysis of this sequence provided a spectrum with a negative peak at λ ∼ 240 nm and two positive peaks at λ ∼ 265 and 290 nm, a signature of hybrid or mixed G4 topology^32^ (Figure 3B left). Because the melting temperature measured at the two positive peaks differed by about 5 °C (Figure 3B right), LTR-FI G4 likely folds into mixed conformations. Indeed, the 1D ^1^H NMR spectrum of LTR-FI G4 in KCl showed several partially overlapped signals in the δ 10.8–12.0 ppm region, a signature of Hoogsteen H-bonded imino protons of G residues, indicating the formation of multiple G4 structures (Figure 3C).

We next moved to assess the LTR G4 binding of the FUS protein from cell extracts by a pull-down assay combined with WB. Immobilized biotinylated oligonucleotides corresponding to LTR-FI G4, LTR-III, LTR-IV, and LTR-II + III + IV G4s and a G-rich sequences unable to fold into G4 as the negative control (Table 2) were incubated with nuclear protein extracts: complexes were cross-linked with formaldehyde and separated by SDS-PAGE. WB assay with an anti-FUS specific antibody showed that FUS tightly bound only to the LTR G4s (Figure 3D, upper panel), because no interaction was observed with control oligonucleotides (Figure 3D, lower panel). Quantification of WB bands indicated that FUS preferential target is LTR-FI G4, which reached 200% of binding compared to the full-length sequence. These data also indicated that in cells LTR-IV is bound almost twofold higher than LTR-III (Figure 3E), in line with the initial pull-down/MS results.

The LTR G4 oligonucleotides were designed with 5T flanking regions, the conformational mobility of which was likely responsible for making the overall oligonucleotide conformation deviate from the classic G4 CD signature (Figure S3A). To assess the impact of the 5T flanking regions on FUS binding, we performed pull-down/WB analysis on LTR-FI and LTR-II + III + IV oligonucleotides lacking the flanking 5Ts. FUS binding to these two oligonucleotides was maintained, with preference for LTR-FI versus LTR-II + III + IV, as also observed in the oligonucleotides with 5T flanking regions: these data indicate that FUS binding is independent of the oligonucleotide flanking regions (Figure S3).

Altogether, the collected data confirm the ability of FUS to induce the folding of an additional previously unreported G4 in the HIV-1 LTR promoter and to tightly bind viral G4 structures, with respect to unstructured DNA.

### FUS Binding to HIV-1 LTR G4s Negatively Regulates Viral Transcription

**Figure 4:**
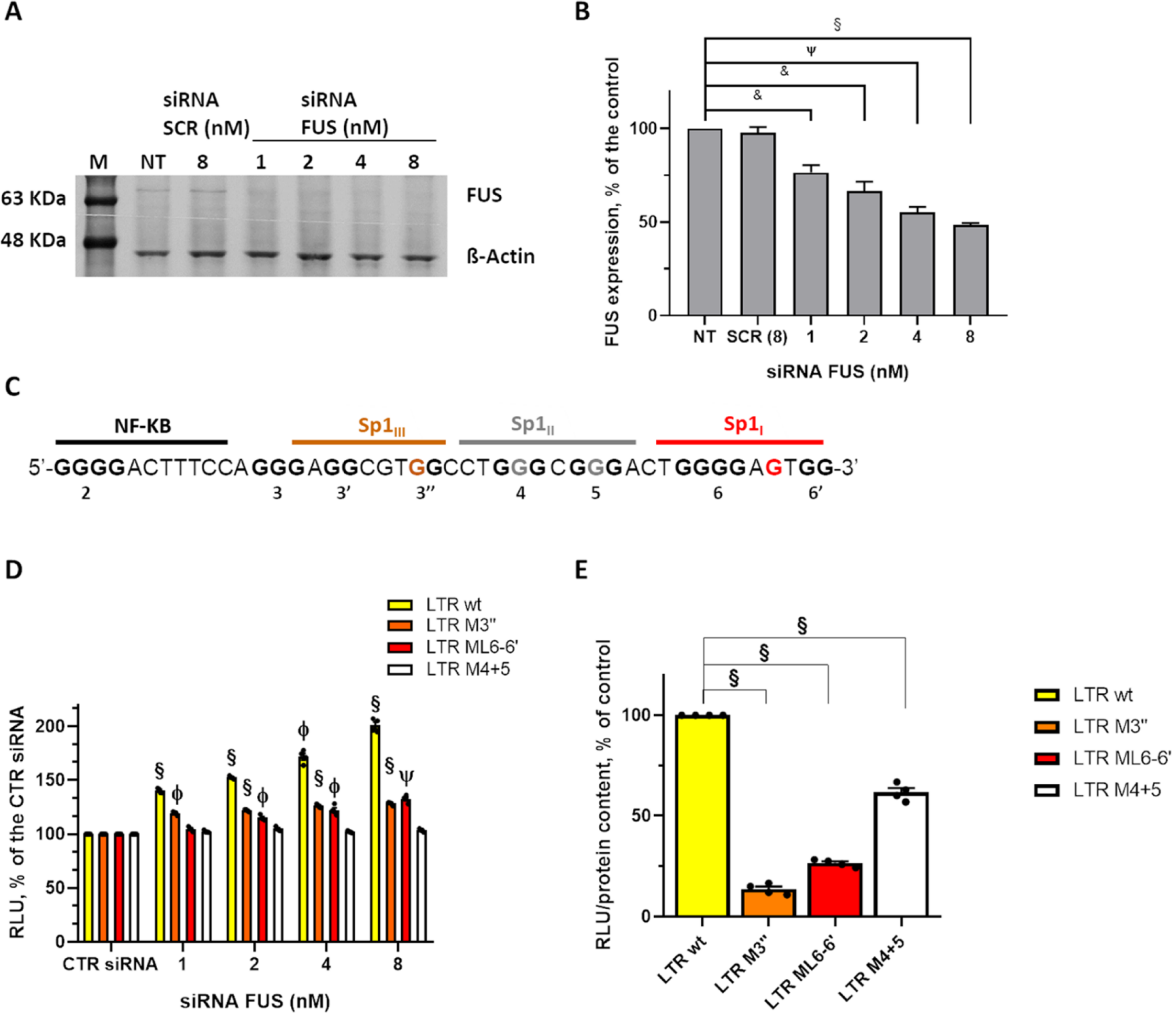
Activity of FUS on the HIV-1 LTR promoter in cells. (A) FUS depletion in HEK 293T cells by FUS siRNAs analyzed by WB with FUS antibody. CTR siRNA indicates scrambled siRNAs used as control. Detection of β-actin was used as control. (B) Quantification of FUS expression levels (average of two independent experiments). Band quantification was normalized on the β-actin signal. *P*-Values (& *P* < 0.05, ψ *P* < 0.005, ϕ *P* < 0.0005, § *P* < 0.0001) and SD are reported. (C) Mutations introduced into the LTR sequence. (D) Analysis of luciferase activity of the wt (yellow bars) *vs* LTR-M3″ (orange bars), LTR-ML6(6′) (red bars), or LTR-M4 + 5 (white bars) promoters in HEK 293T cells transiently transfected with the LTR luciferase plasmids. Luciferase values were normalized on cellular protein levels. Four independent experiments were performed with four replicates per condition in each experiment. A double-tailed *T*-test was performed comparing the transcriptional activity of each LTR-luciferase construct (LTR-wt, LTR-M3″, LTR-ML6-6′, and LTRM4 + 5) during FUS silencing (1–2–4–8 nM siRNA FUS) vs the relative transcriptional activity in the presence of the control siRNA (CTR siRNA). *P*-Values (& *P* < 0.05, ψ *P* < 0.005, ϕ *P* < 0.0005, § *P* < 0.0001) and standard error of the mean (s.e.m.) are reported. (E) Promoter strength analysis of mutated constructs relative to wt. Luciferase values were normalized on cellular protein levels. Mean of two independent experiments with four technical replicates each were performed. *P*-Values (ψ *P* < 0.005, ϕ *P* < 0.0005, § *P* < 0.0001) of double-tailed *T*-test and s.e.m. are reported.

To assess the biological role of FUS in the regulation of HIV-1 transcription, we set up a luciferase reporter assay, in which the luciferase transcription was under the control of the full-length wild-type (wt) or mutated HIV-1 LTR promoter. Three mutated constructs were prepared: ML6(6′), where LTR-IV G4 formation was impaired; M3″, which prevents the formation of LTR-FI; and M4 + 5, unable to fold into any G4 structure, as previously reported.^22,29^ Upon transfection with luciferase plasmids, HEK 293T cells were treated with increasing concentrations of siRNAs designed to specifically target FUS expression. Depletion of FUS was confirmed by WB analysis (Figure 4A). FUS protein was successfully silenced (up to 49% with respect to the control cells, at the highest siRNA concentration, Figure 4B) and siRNA treatment showed dose-dependent FUS depletion. Post-silencing luciferase intensity showed a remarkable, dose-dependent increase of the transcriptional activity of wt LTR, corroborating the ability of FUS to stabilize LTR G4s, therefore inhibiting viral transcription. The luciferase signal was not affected by FUS depletion in the non-G4 M4 + 5 promoter, as expected, confirming the G4-mediated mechanism of action. In ML6(6′) and M3″ templates, a significant increase of transcriptional activity was observed, although to a lower extent compared to wt (Figure 4C). Because mutations located in G-tract 3″ and between G-tracts 6 and 6′ allow the formation of LTR-IV and LTR-FI G4, respectively (Figure 3A), the increase in the luciferase signal is likely due to the stabilization of these two G4s. Note that mutations in these constructs highly modified the LTR basal promotorial strength compared to the wt promoter (Figure 4D) in the absence of siRNAs: this effect likely derives from modifications in binding sites I and III of SP1 (Figure 4B)^33^ and possibly of other yet-to-be-identified proteins.

## Discussion

Viruses are obligate intracellular parasites, which exploit host cell proteins and pathways for their own replication. The integrated HIV-1 5′-LTR promoter, for example, drives viral transcription through the recruitment of cellular proteins: among these, Sp1 and NF-κB TFs, established key elements in HIV-1 promoter activity, interact at their ds consensus sequences in the LTR G-rich region.^9,25^ We have previously shown that the cellular proteins NCL, hnRNP A2/B1, and hnRNP K bind to non-canonical secondary structures in the LTR G-rich region and regulate viral promoter activity.^22,29,30^ Here, we identified FUS as an additional G4-interacting player in viral transcription.

FUS has been reported to bind mainly RNA G4s in human cells^34−37^ through its RGG domain.^38^ FUS interacts with the telomeric repeat-containing RNA, leading to the regulation of histone modifications at telomeres and of telomere length in vivo;^35,39^ it also binds G4s formed in dendritic mRNAs, regulating their translation.^36^ At the DNA level, a recent G4 ChIP-seq analysis found that the recovered sequences were enriched in FUS binding sites and that the protein could directly bind to G4s and to its reported binding consensus sequence at comparable levels.^40^ In HIV-1, FUS has been reported to display an opposite effect on viral transcription: it silences viral transcription through interaction with AFF4 and ELL2, proteins that are involved in transcription elongation processes,^41^ while it enhances viral transcription by binding to an HIV-1-enhanced lncRNA.^42^

Here, we showed that FUS directly binds to G4s that form in the integrated HIV-1 LTR promoter. Interestingly, while the previously identified cellular protein NCL preferentially binds and stabilizes LTR-III, that is, the major G4 that folds in the LTR G-rich region,^22^ FUS preferred less stable G4s, such as LTR-IV and the newly identified LTR-FI. This preference was evident when G4–FUS complexes formed in the cellular context, suggesting that additional factors may help stabilize the least stable G4s and/or favor FUS recognition. Binding of FUS to these LTR G4s hampers polymerase progression and transcription, similar to the activity of NCL, which stabilizes LTR-III,^22^ and opposite to hnRNP A2/B1, which unfolded LTR G4s.^29^ NCL selective binding of LTR-III G4, which carries a long stem loop,^26^ was later rationalized by NCL preferential recognition of G4s containing long loops.^43,44^ In the case of FUS, both LTR-IV and LTR-FI can form bulged G4s, which could be the recognition feature. We thus propose that cell factors stabilize bulged G4s, which in turn are selectively recognized by FUS.

We and other groups have recently shown that G4 folding in the native chromatin context is associated to transcription,^45,46^ while plasmid-based reporter assays such as that used here generally indicate the opposite effect.^25,47^ Because the HIV-1 genome integrates into the host cell chromosome, the chromatin state might be relevant for G4 activity. In the case of HIV-1 LTR, the G-rich region that folds in multiple G4s in vitro is constantly maintained in an open and accessible form, where nucleosomes (Nuc0 and Nuc1) bind at flanking sequences independently of the integration site^48^ and where TFs bind, as demonstrated both in vitro and in vivo.^49,50^ It is thus plausible that in this case the behavior observed in the plasmid assay is preserved also in the chromosome context. In the cell analysis of G4, folding and protein interaction will allow further disclosure on the HIV-1 LTR G4-mediated regulation of viral transcription activity.

HEK 293T was here used to prepare cell extracts. This cell line was chosen because it is both susceptible and permissive to HIV-1 infection^31^ and it is commonly used to produce viral stocks.^51^ We cannot exclude that the use of a different cell line would point out other interacting proteins; however, the HEK 293T gene expression profile is very similar to that of T lymphocytes, the natural HIV-1 cell targets in vivo;^31^ hence, proteins recovered from these cells are likely the most relevant ones.

The effect of FUS binding was not studied in HIV-1-infected cells because FUS has several effectors and effects in the cell. FUS silencing in the cell would combine all these effects, and the outcome at the viral level would not describe the sole inhibition of FUS–LTR G4 interaction. For the very same reason, FUS would not be a good target to block HIV-1 infection, while its G4 target and/or its complex with the G4 target may well be.

Our data indicate that the G4 landscape in the G-rich region of the HIV-1 LTR promoter is even more dynamic and complex than previously envisaged. Even low stability G4s are bound by cellular proteins, with the resulting complexes modulating promoter activity. On the whole, diverse proteins interact at the LTR G4s and exert effects that can range in intensity and direction, for the final fine tuning of viral transcription. These results are in line with a recent observation that numerous TFs are retrieved bound to the same G-rich region in cells.^40^ Because FUS interaction with other cell and viral macromolecules affects HIV-1 transcription in different ways,^41,42^ we propose the LTR G4s to serve as virus sensors of the cell state: through their multiple interactions with cell proteins, LTR G4s sense whether the virus is in a favorable environment to actively transcribe its genes, thus completing its replication cycle and producing new virions, or whether shutting down transcription and remaining in a latent state is the best option for the virus. This observation is further sustained by our previous findings that all human and primate lentiviruses and some retrovirus families as well have a similar LTR G4 organization, that is, with several G-tracts that allow the formation of multiple, mutually exclusive G4s.^9,52^ Thus, the complex and dynamic G4 landscape at the HIV-1 and lenti/retrovirus LTR promoter could be the prime virus center that recognizes cell conditions and consequently dictates the best viral replication strategy.

## Conclusions

Since its discovery in the early eighties, HIV-1 has been one of the most challenging topics in virology. Advances in medicine and biology have taken a stride forward in the management of the virus and its related diseases. However, after almost 40 years of constant efforts, no cure is yet available to eradicate the virus from the infected human host; therefore, identifying innovative and unique antiviral targets is of utmost importance. The present data on FUS interaction at a previously unidentified G4 in the HIV-1 LTR promoter add complexity to the cell-mediated regulation of HIV-1 transcription and further support LTR-G4s and their interaction with cell proteins as sensible innovative targets for the design of antiviral compounds. This could be a feasible direction to develop agents acting at the integrated viral genome, with consequent inhibition of both the replicating and latent viruses. Overall, our data disclose new insights into viral pathogenesis and corroborate the feasibility of an anti-HIV-1 approach based on G4s.

## Methods

### Oligonucleotides and Cells

Oligonucleotides (Table 2) and chemical reagents were purchased from Sigma-Aldrich, Milan, Italy. Human embryonic kidney (HEK) 293T (ATCC # CRL-3216) cells were grown in DMEM (Gibco, Thermo Fisher Scientific, Waltham, MA, USA) supplemented with 10% heat-inactivated fetal bovine serum (Gibco, Thermo Fisher Scientific, Waltham, MA, USA) in a humidified incubator maintained at 37 °C with 5% CO_2_.

### Nuclear Protein Extraction and Pull-Down Assay

Protein nuclear extract from HEK 293T cells was obtained by using the NXTRACT kit (Sigma-Aldrich, Milan, Italy) and quantified using the Pierce BCA protein assay kit (Thermo Fisher Scientific, Monza, Italy), according to manufacturers’ instructions. Biotinylated oligonucleotides were coupled with 30 μL streptavidin-agarose beads (Sigma-Aldrich, Milan, Italy) for 2 h at 37 °C and then incubated with nuclear protein extracts (150 μg) for 2 h at 37 °C in a binding buffer (Tris-HCl 20 mM pH 8, KCl 30 mM, MgCl_2_ 1.5 mM, protease inhibitor cocktail 1%, NaF 5 mM, Na_3_VO_4_ 1 mM, poly[dI-dC] 1.25 ng/μL). After PBS 1× and NaCl (0.2 and 1 M) washes, beads were collected, resuspended in 50 μL Laemmli buffer, and finally incubated at 95 °C for 5 min. Supernatants were separated in 12% SDS-PAGE, and after Coomassie blue staining, gel lanes were cut in ∼0.5 cm pieces, washed with 50% methanol and 2.5% acetic acid, dehydrated with acetonitrile, and then reduced with 30 μL DTT (10 mM in ammonium bicarbonate 100 mM) for 30 min at room temperature. DTT excess was then neutralized by alkylation with 30 μL iodoacetamide (50 mM in ammonium bicarbonate 100 mM) for 30 min at room temperature. Bands were washed with 100 mM ammonium bicarbonate, dehydrated with acetonitrile twice, and then digested overnight with 1 μg MS-grade trypsin (Thermo Fisher Scientific, Waltham, MA, USA) in 50 μL ammonium bicarbonate 50 mM. Peptides were extracted twice with formic acid 5% and twice with acetonitrile 50%/formic acid 5%. The peptide mixture was further desalted in a silica nanocolumn (Polymicro Technologies, Phoenix, AZ, USA) packed in-house with the pinnacle C18 pack material (Thermo Fisher Scientific, Waltham, MA, USA). The desalted mixture was analyzed by direct infusion electrospray ionization on a Thermo Fisher Scientific (Waltham, MA, USA) LTQ-Orbitrap Velos mass spectrometer utilizing quartz emitters produced in-house. A stainless-steel wire was inserted through the back end of the emitter to supply an ionizing voltage that ranged between 0.8 and 1.2 kV. Putative peptides samples were submitted to tandem mass spectrometric (MS/MS) analysis: the masses of the 50 most intense fragment ions were employed to perform a Mascot Database Search to identify their parent protein. Significant Mascot hits were accepted as positive matches, and those with scores lower than 30 were not retained, nor those displaying scores higher than 30 in the interaction with the magnetic streptavidin-coated matrix. The displayed numeric scores were assigned by Mascot software and indicate the probability that the observed protein match is not a random event, and it is strictly related to the number of fragments that match the indicated protein.^53^

### *Taq* Polymerase Stop Assay

*Taq* polymerase stop assay was carried out as previously described.^25^ Briefly, the 5′-end radiolabeled primer (Table 2) was annealed to the DNA template (Table 2, 200 nM) in lithium cacodylate buffer in the absence or presence of KCl 100 mM by heating at 95 °C for 5 min and subsequent gradual cooling to room temperature. Where specified, samples were incubated with purified human FUS (1 μM) at 37 °C for 2 h. Primer extension was then performed using 2U of AmpliTaq Gold DNA polymerase (Applied Biosystems, Carlsbad, CA, USA) for 30 min at 37 or 47 °C, as indicated. Reactions were stopped by ethanol precipitation. Elongation products were separated on a 16% denaturing gel and finally visualized by phosphorimaging. Markers were obtained through the Maxam and Gilbert sequencing protocol.^54^

### Circular Dichroism

Oligonucleotides were diluted to a final concentration of 3 μM in phosphate buffer (20 mM, pH 7.4) and KCl 100 mM. Samples were heated at 95 °C for 5 min and then slowly cooled to room temperature overnight. CD spectra were recorded on a Chirascan-Plus (Applied Photophysics, Leatherhead, UK) equipped with a Peltier temperature controller using a quartz cell with a 5 mm optical-path length. Thermal unfolding experiments were recorded from 230 to 320 nm over a temperature range of 20–90 °C. Acquired spectra were baseline-corrected for signal contribution from the buffer, and the observed ellipticities were converted to mean residue ellipticity according to θ = deg·cm^2^/dmol (molar ellipticity). *T*_m_ values were calculated according to the van’t Hoff equation applied for a two-state transition from a folded state to an unfolded state, using SigmaPlot software (Systat Software, San Jose, CA, USA).

### NMR Spectroscopy

Oligonucleotide for NMR measurements was synthesized on a K&A Laborgeräte GbR DNA/RNA synthesizer H-8 using standard phosphoramidite chemistry in the DMT-on mode. Oligonucleotide was cleaved from support and deprotected with 1:1 mixture of methylamine and ammonium hydroxide. Glen-Pak cartridges in reverse-phase mode were used to remove non-full-length abortive sequences. Oligonucleotide was further desalted with FPLC, dried on a lyophilizer, and dissolved in water at 1 mM concentration. pH was adjusted to neutral with LiOH, and potassium phosphate buffer (pH 7.4) and potassium chloride were added to the final concentration of 20 and 80 mM, respectively. Oligonucleotide was annealed at 95 °C for 5 min and left for cooling down to room temperature over a course of several hours. NMR experiments were performed at 25 °C on a Bruker AVANCE NEO 600 MHz NMR spectrometer equipped with a 5 mm ^1^H-optimized quadruple resonance cryo probe. Spectra were processed and visualized with TopSpin 4.08.

### G4-Binding Protein Cross-Linking Assay

Protein nuclear extracts were obtained as described in the pull-down experiment. Biotinylated oligonucleotides (150 pmol) were folded in phosphate buffer 100 mM pH 7.4 and KCl 100 mM and bound to streptavidin-coated magnetic beads 50 μL. DNA coupled beads were incubated with nuclear proteins extract (50 μg) at 4 °C for 90 min and excess protein was washed with Tris-HCl 50 mM pH 7.5—NaCl 150 mM. Samples were fixed with formaldehyde 5% in PBS for 30 min at room temperature, washed, and then analyzed by WB analysis, with an anti-FUS antibody (mouse monoclonal 4H11; Santa Cruz Biotechnology, Dallas, TX, USA). Briefly, samples were electrophoresed on a 10% SDS-PAGE and transferred to a nitrocellulose blotting membrane (Amersham Protan, GE Healthcare Life Science, Milan, Italy) by using a trans-blot SD semi-dry transfer cell (Bio-Rad Laboratories, Milan, Italy). The prestained protein marker VI (10–245) (AppliChem, Darmstadt, Germany) was used as the molecular weight ladder. The membrane was blocked with 5% skim milk in the PBS solution and incubated with the anti-FUS primary antibody and then with the ECL Plex Goat-α-Mouse IgG-Cy5 (GE Healthcare Life Sciences, Milan, Italy). Images were captured on Typhoon FLA 9000. Bands were quantified using ImageQuant TL software (GE Healthcare Europe, Milan, Italy). *P* values were calculated employing the unpaired two-tailed Student’s *t*-test, and the significance for each sample (**, ***) has been indicated according to the calculated *P* value. Apparent *K*_D_ was calculated as the concentration at which the half-saturation was reached.

### siRNA and Luciferase Reporter Assay

SiRNA trilencer targeting human FUS and a scrambled negative control duplex were purchased from OriGene Technologies (SR301670, Rockville, MD, USA). HEK 293T cells were transfected with human FUS siRNA or control siRNA at indicated concentrations using Lipofectamine RNAiMAX (Invitrogen, Thermo Fisher Scientific, Waltham, MA, USA) according to the manufacturer’s instructions. Silencing efficiency was confirmed through WB analysis, as described above, using anti-FUS and anti-β-actin (Sigma-Aldrich, Milan, Italy) primary antibodies. pLTR luciferase wt or mutated plasmids were transfected into the same cells 24 h post-silencing using Lipofectamine 3000 (Invitrogen, Thermo Fisher Scientific, Waltham, MA, USA). In the transfected cells, the LTR promoter activity was assessed as a firefly luciferase signal, measured by the Britelite plus reporter gene assay system (PerkinElmer Inc., Milan, Italy) following the manufacturer’s instructions. The luciferase signal was measured by a Victor X2 multilabel plate reader (PerkinElmer Italia, Milan, Italy). Each assay was performed in duplicate, and each set of experiments was repeated at least three times. The signal was normalized to the total protein content, determined by the BCA assay, which was performed after cell lysis and protein extraction.

### Statistics and Data Analysis

All *P* values were calculated using an unpaired two-tailed Student’s *t*-test, and the significance for each sample was indicated using the calculated *P* value. *P* values were not calculated for data sets with *n* < 3. All the statistical analyses were performed using GraphPad Prism 8. The error bars indicate s.d. or s.e.m., as stated in the figure legends. Image quantification was performed using ImageJ software unless otherwise stated.

## Supporting Information Available

WB, CD spectra, and pull-down/WB assay (PDF)

## Abbreviations

HIV-1: human immunodeficiency virus type 1
AIDS: acquired immunodeficiency syndrome
ds: double-stranded
G4: G-quadruplex
G: guanine
PQS: putative quadruplex sequence
NCL: nucleolin
RNP: ribonucleoprotein
LTR: long terminal repeat
TF: transcription factor
FUS: fused in liposarcoma
PAGE: polyacrylamide gel electrophoresis
CD: circular dichroism
NMR: nuclear magnetic resonance
wt: wild-type
