# Supplementary material for: Fused in Liposarcoma Protein, a New Player in the Regulation of HIV-1 Transcription, Binds to Known and Newly Identified LTR G-Quadruplexes
Source: ACS Infect Dis. 2022 May 3;8(5):958–68. doi: 10.1021/acsinfecdis.1c00508 (PMC9112328; doi:10.1021/acsinfecdis.1c00508)
Supplement: Supplementary file 1 — id1c00508_si_001.pdf [file id1c00508_si_001.pdf]

Supporting Information

Fused in Liposarcoma (FUS) protein, a New Player in the Regulation of HIV-1 Transcription, binds to Known and Newly identified LTR G-quadruplexes

Emanuela Ruggiero,[†,#] Ilaria Frasson,[†,#] Elena Tosoni,[†] Matteo Scalabrin,[†] Rosalba Perrone,[‡] Maja Marušič,[§] Janez Plavec[§] and Sara N. Richter*,[†].

[†]Department of Molecular Medicine, University of Padua, via Aristide Gabelli 63, 35121 Padua, Italy

[‡]Buck Institute for Research on Aging, 8001 Redwood Boulevard, Novato, California 94945, United States

[§]Slovenian NMR center, National Institute of Chemistry, Hajdrihova, 19, Ljubljana SI-1000, Slovenia

Content:

Figure S1 S2

Figure S2 S3

Figure S3 S4

A

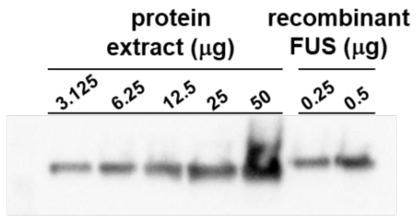

B

	ug	Band intensity	FUS amount in the extract (μg)
**NE 293T**	3.125	3161.96	0.30
	6.25	4145.60	0.37
	12.5	4371.05	0.39
	25	5956.05	0.51
	50	out of range	nd
**FUS**	0.25	2625.91	
	0.5	6005.10	

C

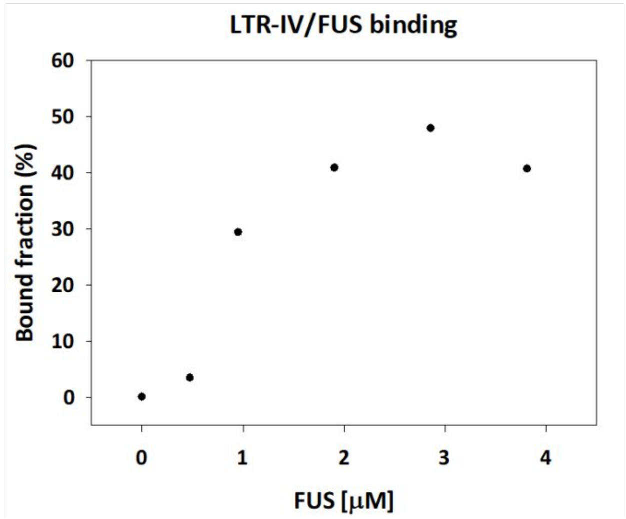

Figure S1. WB analysis of FUS protein from cell protein extracts. A) The amount of FUS in the cell protein extract was calculated comparing band intensities with those of a purified recombinant protein. B) Quantification of WB band intensities from panel A). C) Plot of the bound fraction of LTR-IV versus increasing concentrations of the native FUS protein from cell protein extracts (see Figure 2A). The apparent K_D was calculated as the concentration at which half saturation is reached.

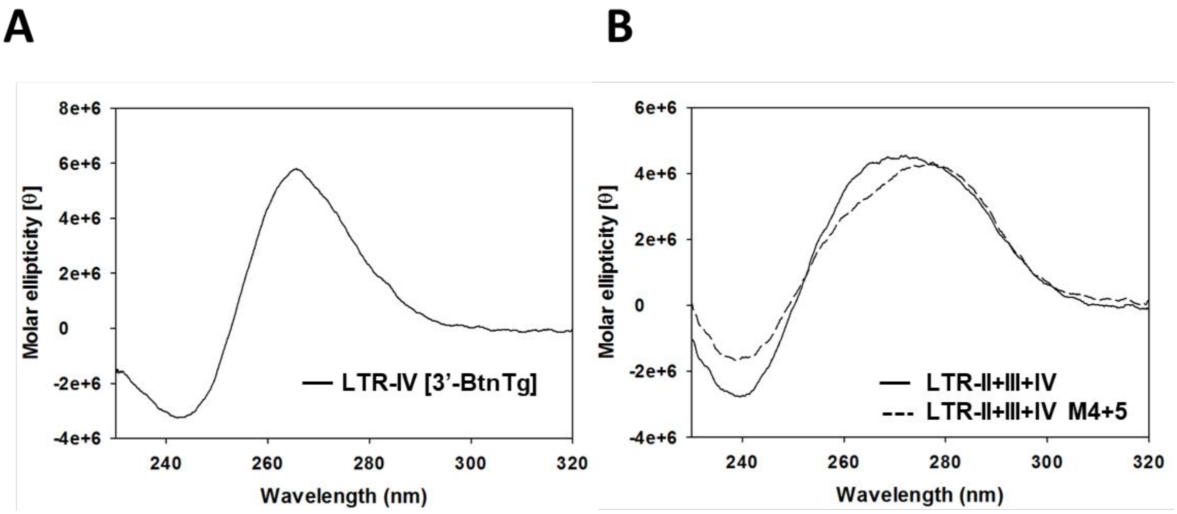

Figure S2. Circular Dichroism analysis of LTR sequences. A) CD spectrum of LTR-IV [3'-BtnTg] sequence used for pull-down experiments. B) CD spectra of LTR-II+III+IV (plain line) and the mutated LTR-II+III+IV M4+5 (dashed line) used for *Taq* polymerase stop assay.[θ] = deg×cm^2/dmol.

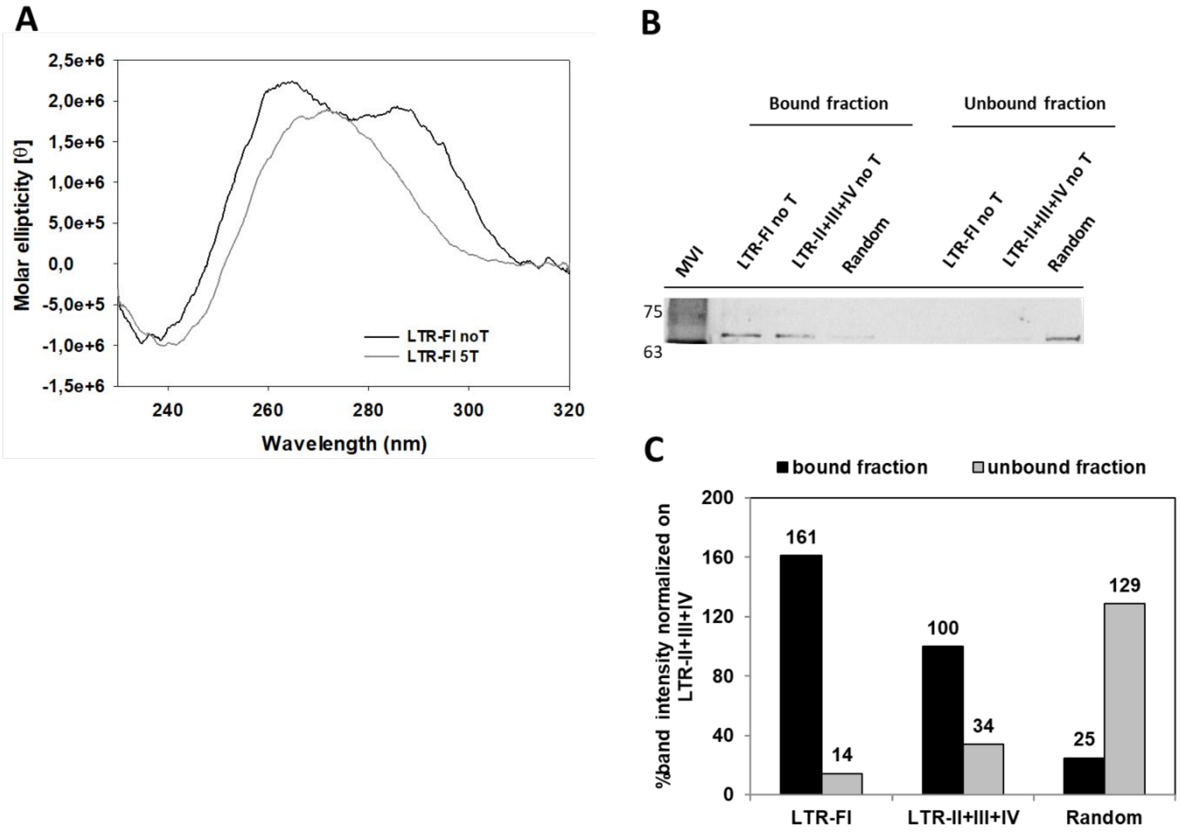

Figure S3. FUS binding to HIV-1 LTR-FI G4 in the absence of flanking regions. A) CD spectra of LTR-FI in the absence and presence of 5T flanking regions. [θ] = deg×cm^2/dmol. B) WB analysis with the anti-FUS antibody, following pull-down and crosslinking. Bound and unbound fractions are reported. MVI is the molecular weight ladder lane and numbers on the left indicate KDa. C) WB bands quantification from panel (B) reported as percentage relative to LTR-II+III+IV G4.
